# Development of a Novel Nanoclay-Doped Hydrogel Adsorbent for Efficient Removal of Heavy Metal Ions and Organic Dyes from Wastewater

**DOI:** 10.3390/gels11040287

**Published:** 2025-04-14

**Authors:** Hang Zhao, Mengmeng Xie, Siyu He, Saishi Lin, Shige Wang, Xiuying Liu

**Affiliations:** 1 School of Chemistry and Chemical Engineering, Wuhan Textile University, Wuhan 430200, China; 2 School of Materials and Chemistry, University of Shanghai for Science and Technology, No. 334 Jungong Road, Shanghai 200093, China; 3 Key Laboratory of Textile Fiber and Products, Ministry of Education, Wuhan Textile University, Wuhan 430200, China

**Keywords:** nanoclay, hydrogel, adsorbent, heavy metal ion, dye, wastewater

## Abstract

Rapid industrialization has led to significant environmental challenges, particularly in wastewater treatment, where the removal of heavy metal ions and organic dyes is critical. This study presents the synthesis and characterization of a high-performance hydrogel adsorbent, (nanoclay)_x_@poly-γ-glutamic acid (γ-PGA)/polyethyleneimine (PEI) hydrogel adsorbent (denoted as N_x_PP, x = 0, 20, 40, 60, and 80), for the efficient removal of heavy metal ions (Cu^2+^, Fe^3+^, and Zn^2+^) and organic dyes (Methylene blue, as a typical example) from wastewater. The hydrogel was prepared using a one-pot method, combining γ-PGA and PEI with varying amounts of nanoclay. The N_80_PP hydrogel demonstrated exceptional adsorption capacities, achieving 224.37 mg/g for Cu^2+^, 236.60 mg/g for Fe^3+^, and 151.95 mg/g for Zn^2+^ within 30 min, along with 88.18 mg/g for Methylene blue within 5 h. The incorporation of nanoclay significantly enhanced the mechanical properties, with compressive strength reaching 560.49 kPa. The hydrogel exhibited excellent reusability, maintaining high adsorption capacity after five cycles. The adsorption kinetics followed a pseudo-second-order model, and the isotherms fit the Freundlich model, indicating a multilayer adsorption mechanism. This study highlights the potential of N_x_PP hydrogels as a versatile and sustainable solution for wastewater treatment.

## 1. Introduction

Wastewater treatment has become a global issue with the rapid industrialization of human society [1]. Organic dyes and heavy metal ions are two of the most common pollutants in wastewater [2]. Organic dyes are widely used in industries such as printing, painting, textiles, and food production [3,4,5,6]. However, many dyes are toxic, carcinogenic, and non-biodegradable, posing significant environmental pollution and severe threats to human health [7,8]. Heavy metal ions in industrial wastewater can accumulate in the food chain, ultimately causing severe health problems for humans [9,10]. Therefore, the efficient removal of organic dyes and heavy metal ions from wastewater is essential for safeguarding environmental sustainability and public health.

Currently, the primary methods for removing organic pollutants and heavy metal ions from wastewater include electrochemical treatment, advanced oxidation processes, coagulation, membrane separation, and adsorption [11,12,13]. Among these methods, adsorption is regarded as one of the most effective approaches for removing organic dyes and heavy metal ions from wastewater [14]. Compared to other techniques, adsorption offers advantages such as low cost, by-product-free operation, ease of implementation, and scalability. Activated carbon is one of the most widely used adsorbents, with good removal efficiency for heavy metal ions and organic waste in wastewater. However, its application in wastewater treatment is significantly constrained by its high cost and the need for regeneration at temperatures exceeding 500 °C [15]. Research has shown that the choice of adsorbent is key to determining the effectiveness and efficiency of adsorption [16]. An ideal adsorbent should possess wide applicability, high adsorption capacity, rapid adsorption kinetics, excellent regeneration performance, and cost-effectiveness [17].

In recent years, researchers have successfully developed a range of novel adsorbents for the removal of organic dyes and heavy metal ions from wastewater [18]. Among these, hydrogels have gained significant attention due to their abundant functional groups, porous structure, ease of preparation, and convenient separation [19,20]. For example, Cai et al. developed a self-healing hydrogel for Cr^6+^ adsorption using hydroxypropyl chitosan, polyacrylamide, and polyvinyl alcohol [21]. This hydrogel is cross-linked by a triple network of dynamic Schiff base bonds, borate bonds, and hydrogen bonds, with a maximum compressive strength of 267 kPa. The results showed that the hydrogel showed a high adsorption capacity for Cr^6+^, indicating its potential for heavy metal adsorption. In another study, Wu et al. developed a double-network composite hydrogel adsorbent with outstanding mechanical properties, reusability, and high adsorption capacity [22]. This hydrogel features a primary network of polyacrylic acid cross-linked with methylene bisacrylamide and a secondary network of polyethylene amine cross-linked with chitosan. The synergistic effect of these double networks enhances the mechanical strength and adsorption efficiency of the composite hydrogel. Despite these advancements, the development of multifunctional adsorbents capable of efficiently removing both organic dyes and heavy metal ions while maintaining excellent mechanical properties, high adsorption capacity, and long-term reusability remains a critical challenge. Moreover, the incorporation of nanomaterials, such as nanoclays, into hydrogel matrices has shown promising potential in enhancing adsorption performance and structural stability [23,24]. However, systematic studies on the synergistic effects of nanoclays and polymeric networks in hydrogel adsorbents are still limited.

In this study, we report the design and fabrication of a novel nanoclay-doped γ-PGA/PEI hydrogel adsorbent (N_x_PP) with enhanced adsorption capacity, superior mechanical strength, and excellent reusability for the simultaneous removal of heavy metal ions and organic dyes from wastewater. The incorporation of nanoclays reinforces the hydrogel structure, improving the material’s performance. The adsorption kinetics, isotherms, and mechanisms were thoroughly investigated to provide a deeper understanding of the adsorption process. This work contributes to the advancement of high-performance, multifunctional hydrogel adsorbents for sustainable wastewater treatment applications.

## 2. Results and Discussion

### 2.1. Synthesis and Characterization of N_x_PP Hydrogels

**Figure 1:**
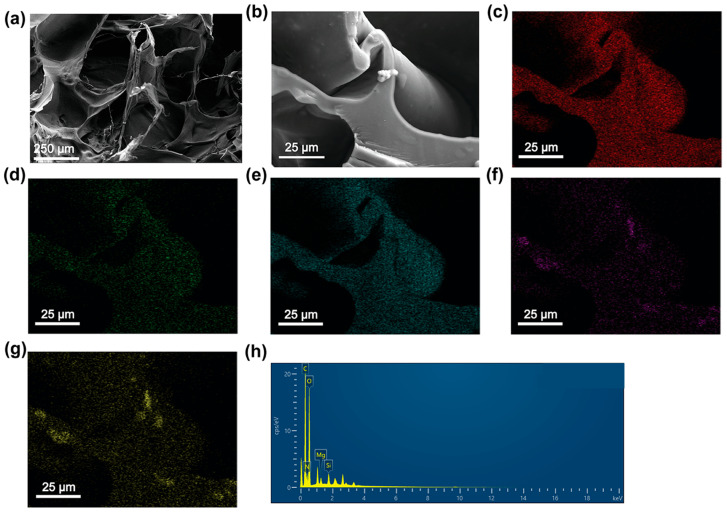
Characterization of N_80_PP hydrogel. (**a**,**b**) SEM image of N_80_PP hydrogel. (**c**–**g**) Elemental mapping for C, N, O, Mg, and Si, respectively. (**h**) EDS of N_80_PP hydrogel.

The N_x_PP hydrogels were synthesized through the cross-linking of γ-PGA with PEI via amide bonds, using 1-Ethyl-3-(3-dimethylaminopropyl)carbodiimide (EDC) and N-hydroxysuccinimide (NHS) as activators to activate the carboxyl groups of γ-PGA. The formation of the hydrogel was first investigated using Fourier transform infrared (FTIR) spectroscopy (Figure S1). All hydrogels exhibited a distinct absorption peak at 1452 cm^−1^, which corresponds to the stretching vibrations of carboxyl groups, confirming their presence in the hydrogel structure. The absorption band at 788 cm^−1^ is attributed to the rocking or wagging vibrations of CH_2_ groups, while the peak at 722 cm^−1^ is characteristic of the out-of-plane rocking vibrations of long, linear alkyl chains (e.g., -(CH_2_)_n_-). In addition, prominent peaks were observed at 1642 cm^−1^ and 1576 cm^−1^, corresponding to the stretching vibration of the amide I band and the bending (scissoring) vibration of the amide II band, respectively, indicating the formation of amide bonds within the hydrogel network. The microstructure of N_80_PP hydrogel was observed through SEM (Figure 1a,b). SEM images revealed that the N_80_PP hydrogel exhibited a loose and porous structure, which could facilitate faster solvent exchange and enhanced ion adsorption capacity. Elemental mapping (Figure 1c–g) suggested the co-existence of C, N, O, Mg, and Si in the hydrogel. Energy Dispersive Spectroscopy (EDS) analysis (Figure 1h) further confirmed the successful incorporation of nanoclay and the effective preparation of N_80_PP hydrogel.

### 2.2. Swelling Properties of N_x_PP Hydrogels

**Figure 2:**
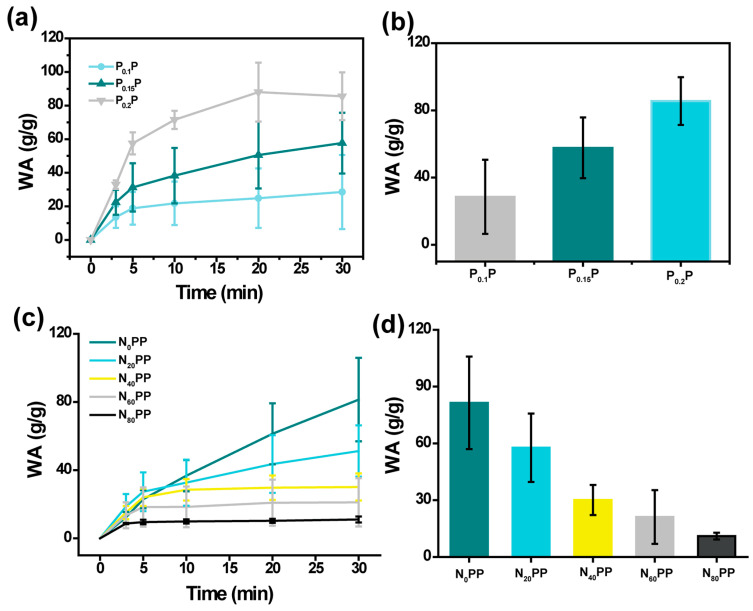
(**a**) Dynamic swelling and (**b**) equilibrium adsorption capacity of P_x_P hydrogels. (**c**) Dynamic swelling and (**d**) adsorption capacity of N_x_PP hydrogels (adsorption duration: 30 min).

The swelling behavior of P_x_P and N_x_PP hydrogels in deionized water at room temperature was measured, showing excellent swelling capacities for both hydrogels. The WA of P_x_P hydrogels increased with the γ-PGA content (Figure 2a), reaching equilibrium within 20 min (P_0.1_P: 28.53 g/g, P_0.15_P: 57.70 g/g, and P_0.2_P: 85.55 g/g, Figure 2b). The enhancement of equilibrium adsorption capacity can be ascribed to the hygroscopic nature of γ-PGA, whereby a higher γ-PGA content leads to an increased WA value. However, the WA of N_x_PP hydrogels decreased as the nanoclay content increased (Figure 2c), with swelling rates of 81.40, 51.22, 30.11, 21.11, and 11.0 g/g for N_0_PP, N_20_PP, N_40_PP, N_60_PP, and N_80_PP hydrogels (Figure 2d), respectively, after 30 min of immersion in deionized water. This decrease in WA is likely due to increased pore density caused by the incorporation of nanoclay.

### 2.3. Mechanical Properties of N_x_PP Hydrogels

**Figure 3:**
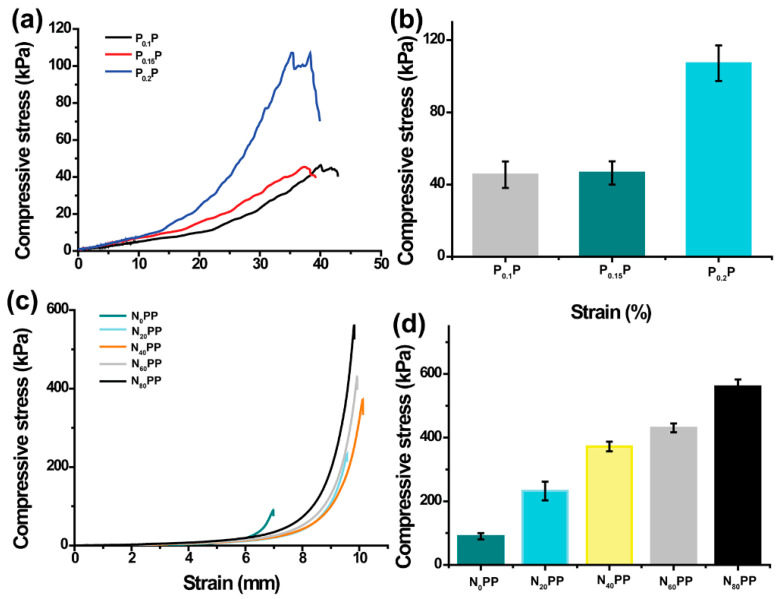
(**a**,**b**) Compressive properties of P_x_P hydrogels; (**c**,**d**) Compressive properties of N_x_PP hydrogels.

While different hydrogel adsorbents demonstrate remarkable adsorption capacities for heavy metal ions, they often suffer from inherent mechanical deficiencies, particularly structural degradation during cyclic adsorption–desorption processes [25,26]. The mechanical performance of P_x_P and N_x_PP hydrogels was systematically investigated through comprehensive compressive strength analysis. Notably, the integration of nanoclay into the hydrogel matrix significantly improved the mechanical robustness of N_x_PP hydrogels, addressing this critical limitation. As shown in Figure 3a,b, the compressive strength of P_x_P hydrogels increased with the γ-PGA content (P_0.1_P: 45.44 kPa, P_0.15_P: 46.40 kPa, and P_0.2_P: 107.18 kPa), possibly due to the increased cross-linking density. With the incorporation of nanoclay, the compressive strength of N_x_PP hydrogels significantly improved (N_0_PP: 89.87 kPa, N_20_PP: 232.3 kPa, N_40_PP: 372.13 kPa, N_60_PP: 430.68 kPa, and N_80_PP: 560.49 kPa, Figure 3c,d). These results indicate that the addition of nanoclay can substantially enhance the compressive strength of the hydrogels, making N_x_PP hydrogels promising candidates with superior mechanical properties. The enhancement in compressive strength through nanoclay incorporation can be attributed to the high aspect ratio of nanoclay particles, facilitating effective stress transfer throughout the composite structure, preventing localized stress concentration. However, the specific reasons still need to be further analyzed.

### 2.4. Cu^2+^ Adsorption Capacity of P_x_P Hydrogels and N_x_PP Hydrogels

**Figure 4:**
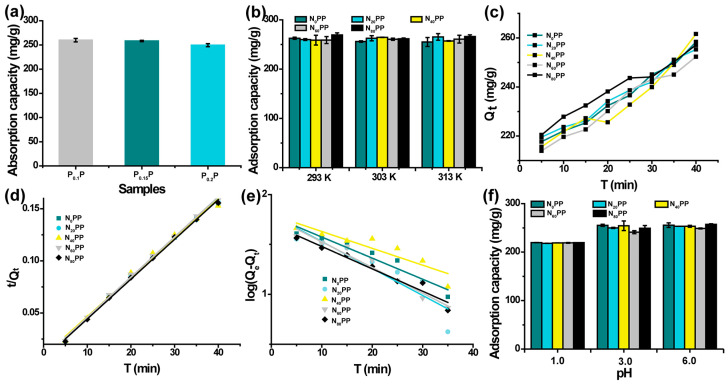
(**a**) Cu^2+^ adsorption capacity of P_x_P hydrogels. (**b**) Cu^2+^ adsorption capacity of N_x_PP hydrogels at different temperatures. (**c**) Cu^2+^ adsorption kinetics of N_x_PP hydrogels. (**d**) Pseudo-first-order Cu^2+^ adsorption kinetics of N_x_PP hydrogels. (**e**) Pseudo-second-order Cu^2+^ adsorption kinetics of N_x_PP hydrogels. (**f**) Effect of different pH on the Cu^2+^ adsorption capacity of N_x_PP hydrogels.

Adsorption experiments were first conducted to evaluate the Cu^2+^ adsorption capacity of PxP hydrogels (pH: 6.0, temperature: 298 K, hydrogel: 30 mg, time: 30 min). As shown in Figure 4a, P_0.1_P, P_0.15_P, and P_0.2_P hydrogels exhibited high Cu^2+^ adsorption capacities (P_0.1_P: 259.84 mg/g, P_0.15_P: 258.17 mg/g, P_0.2_P: 245.59 mg/g), with minimal differences between the three samples. To investigate the effect of temperature on the adsorption capacity of N_x_PP hydrogels, we measured the adsorption performance of N_x_PP hydrogels for Cu^2+^ at different temperatures (293 K, 303 K, and 313 K, Figure 4b). The N_x_PP hydrogels exhibited excellent adsorption capacity for Cu^2+^ at all tested temperatures. However, the adsorption amounts showed minimal variation across the three temperatures, with the N_80_PP hydrogel adsorbing 269.32 mg/g at 293 K, 261.64 mg/g at 303 K, and 266.20 mg/g at 313 K. Similarly, although adsorption equilibrium was not reached, the adsorption kinetics of Cu^2+^ onto N_x_PP hydrogels with varying nanoclay contents exhibited minimal differences (Figure 4c). Adsorption capacities after 30 min were 258.38 ± 6.01, 225.30 ± 4.85, 261.60 ± 1.15, 252.33 ± 11.05, and 257.12 ± 5.28 mg/g for N_0_PP, N_20_PP, N_40_PP, N_60_PP, and N_80_PP hydrogels, respectively. These results demonstrate the high efficiency and rapid Cu^2+^ adsorption capabilities of N_x_PP hydrogels. The adsorption process was further investigated by comparing the actual Cu^2+^ adsorption kinetics with pseudo-first-order and pseudo-second-order models (Figure 4d,e, Table 1). The kinetic curves were better fitted by the pseudo-second-order model, with correlation coefficients (R^2^) of 0.996, 0.997, 0.991, 0.998, and 0.998 for N_0_PP to N_80_PP hydrogel, indicating that the adsorption behavior likely involves chemisorption.

### 2.5. Effect of pH and Temperature on N_x_PP Hydrogels’ Adsorption Capacity

The adsorption capacity of N_x_PP hydrogels for Cu^2+^ in deionized water was studied as a function of pH. From pH 1.0 to 6.0, the adsorption capacity increased (Figure 4f). At pH 6.0, the adsorption capacity reached its maximum (N_80_PP: pH = 1.0, 219.51 mg/g; pH = 3.0, 249.16 mg/g; pH = 6.0, 257.23 mg/g). The increase in adsorption capacity with pH is attributed to the enhanced chelation affinity between amine (-NH_2_) groups and metal ions and the increased cation exchange effect between hydroxyl (-OH) groups and metal ions. Even at pH 1.0, the adsorption capacity remained as high as 219.51 mg/g, suggesting the potential of this hydrogel for industrial wastewater treatment under harsh conditions.

### 2.6. Adsorption Isotherms of N_x_PP Hydrogels

**Figure 5:**
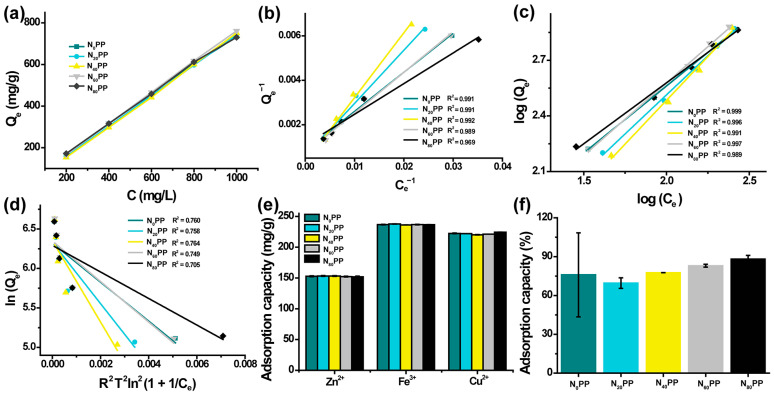
(**a**) Adsorption of Cu^2+^ by N_x_PP hydrogels at different initial concentrations. (**b**) Langmuir, (**c**) Freundlich, and (**d**) Dubinin-Radushkevich isotherm models for the adsorption behavior of N_x_PP hydrogels. (**e**) Adsorption of various metal ions by N_x_PP hydrogels and (**f**) Adsorption of MB by N_x_PP hydrogels.

To further explore the underlying mechanism of temperature effects on the adsorption capacity of N_x_PP hydrogels, we studied the thermodynamic properties of the hydrogel based on adsorption thermodynamics and Gibbs free energy equations. At 293 K, the adsorption performance of the hydrogel for Cu^2+^ was evaluated in wastewater solutions with varying initial Cu^2+^ concentrations (Figure 5a). The adsorption thermodynamic mechanism was further investigated using Langmuir, Freundlich, and Dubinin-Radushkevich isotherm models, with corresponding linear fitting shown in Figure 5b–d and Table 2. The results indicate that the adsorption of Cu^2+^ by N_x_PP hydrogels better follows the Freundlich isotherm model, as the correlation coefficients (R^2^) for N_0_PP, N_20_PP, N_40_PP, N_60_PP, and N_80_PP hydrogels were 0.999, 0.997, 0.991, 0.997, and 0.989, respectively.

### 2.7. Adsorption Capacity of N_x_PP Hydrogels for Different Metal Ions

We evaluated the adsorption performance of N_x_PP hydrogels for various metal ions (Cu^2+^, Zn^2+^, Fe^3+^) under controlled conditions (pH 6.0, temperature 298 K, hydrogel mass 30 mg, adsorption time 30 min) to assess their versatility in adsorbing metal ions. As shown in Figure 5e, N_x_PP hydrogels exhibited adsorption for all three selected metal ions, with the highest adsorption observed for Fe^3+^. The N_80_PP hydrogel adsorbed 236.60 mg/g of Fe^3+^, while the adsorption for Zn^2+^ was the lowest at 151.95 mg/g, and for Cu^2+^, the adsorption reached 224.37 mg/g, nearly equal to that of Fe^3+^. The adsorption is higher than chitosan composites (<120 mg/g for Cu^2+^ [27]) and crosslinked carboxymethyl Sago starch/citric acid hydrogel (<20 mg/g for Zn^2+^ [28]). However, the adsorption differences among the N_x_PP hydrogels for the same metal ion were minimal. The highest adsorption capacity for Fe^3+^ in N_x_PP hydrogels may be attributed to its elevated charge density and robust coordination with amino/hydroxyl groups. Therefore, N_x_PP hydrogel adsorbents can be applied to the adsorption of various heavy metal ions.

### 2.8. Adsorption Capacity of N_x_PP Hydrogels for (Methylene Blue) MB

To assess the adsorption performance of N_x_PP hydrogels for organic dyes, we measured the adsorption of MB in wastewater using UV-Vis spectrophotometry. The adsorption behavior of N_x_PP hydrogels for MB over 5 h was evaluated. The increased nanoclay content enhances the hydrogel’s mechanical strength and thereby provides additional adsorption sites through synergistic interactions, including electrostatic attraction, hydrogen bonding, and π-π stacking, leading to higher MB adsorption efficiency. As shown in Figure 5f, after 5 h, the percentage adsorption of MB by N_0_PP, N_20_PP, N_40_PP, N_60_PP, and N_80_PP hydrogels was 75.98%, 69.57%, 77.62%, 82.97%, and 88.18%, respectively. These results suggest that the prepared hydrogels can be utilized for the adsorption of both organic dyes and heavy metal ions, demonstrating the potential of this hydrogel for the removal of pollutants from wastewater.

### 2.9. Adsorption Mechanism of N_x_PP Hydrogels

**Figure 6:**
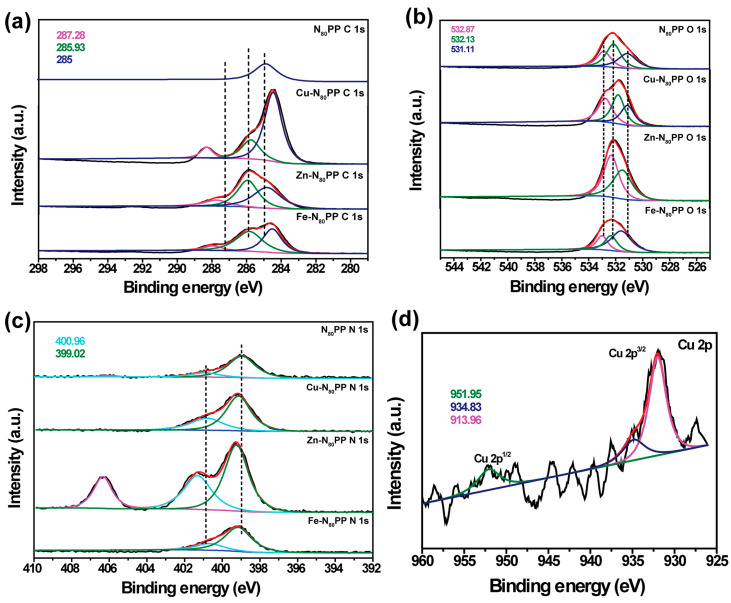
(**a**) C 1s XPS spectra of the N_x_PP hydrogels before and after Cu^2+^ adsorption. (**b**) O 1s XPS spectra of the N_x_PP hydrogels before and after Cu^2+^ adsorption. (**c**) N 1s XPS spectra of the N_x_PP hydrogels before and after Cu^2+^ adsorption. (**d**) XPS spectrum of Cu 2p after the adsorption of Cu^2+^.

To investigate the adsorption mechanisms of N_x_PP hydrogels for heavy metal ions, we employed Cu^2+^ as a representative and XPS to determine the chemical composition of N_80_PP hydrogel before and after the adsorption of Cu^2+^. The C 1s XPS spectrum of the hydrogel prior to adsorption appeared at 278.28 eV (C-H/C-C), 285.93 eV (C-N/C-O), and 285 eV (O-C-O) (Figure 6a). The O 1s spectrum appeared at 532.87 eV (O-C-O), 532.13 eV (C-O-C), and 531.11 eV (C-O-H) (Figure 6b). The N 1s spectrum revealed two fitting peaks: -NH_3+_ (400.96 eV) and -NH_2_/-NH (399.02 eV) (Figure 6c). Following the adsorption of heavy metal ions, Cu^2+^ interacts with the lone pairs of electrons on the oxygen or nitrogen atoms of -OH or -NH_2_/-NH. This interaction leads to a reduction in the electron density of -OH or -NH_2_/-NH, resulting in a peak shift to higher binding energies [29]. Simultaneously, after Cu^2+^ adsorption, the fitting peak of C-O-H in O 1s and the fitting peak of -NH_2_/-NH in N 1s both shifted to higher binding energies, confirming the presence of coordination interactions [27]. As shown in Figure 6d, the XPS spectrum of Cu 2p displays three peaks at 951.95 eV, 934.83 eV, and 913.96 eV, with the binding energies at 951.95 eV and 913.96 eV corresponding to the Cu 2p_1/2_ and Cu 2p_3/2_ orbitals, respectively. In summary, the adsorption mechanism can be summarized as chelation interactions between -NH_2_ and -NH with metal ions.

### 2.10. Reusability of N_x_PP Hydrogels

The reusability of hydrogel adsorbents is crucial for cost-effective environmental protection. We evaluated the reusability of N_x_PP hydrogels for heavy metal ion adsorption using Cu^2+^ as a model, performing multiple adsorption–desorption cycles. After each adsorption cycle, the hydrogels were placed in HCl and agitated to facilitate the desorption. After desorption, the hydrogels completely lost their characteristic blue color, indicating that the majority of Cu^2+^ ions were effectively removed during the regeneration process. The adsorption capacity of the hydrogel slightly decreased after five cycles, with N_0_PP, N_20_PP, N_40_PP, N_60_PP, and N_80_PP hydrogels showing adsorption capacities of 218.43 mg/g, 219.98 mg/g, 217.35 mg/g, 220.89 mg/g, and 221.41 mg/g, respectively, in the fifth cycle. However, no significant difference was detected during these five cycles. Further recovery and reusability activity results demonstrate that all hydrogel samples exhibited excellent reusability and stability. Notably, N80PP retained 96.9% ± 1.2% of its initial adsorption capacity after 5 cycles (Table 3). These results demonstrate that N_x_PP hydrogel adsorbents were regenerated after desorption, freeze-drying, and could be reused for subsequent adsorption–desorption cycles. It is worth noting that the incorporation of nanoclay enhanced the mechanical properties of the hydrogel (increasing the compressive strength from 89.87 kPa (N_0_PP) to 560.49 kPa (N_80_PP)), which may also support its durable usages.

## 3. Conclusions

Hydrogels have been extensively used for water treatment and other applications [30,31]. In this study, we successfully developed a novel, cost-effective, and highly efficient N_x_PP hydrogel adsorbent doped with nanoclay for the removal of heavy metal ions and organic dyes from wastewater. Within 30 min, the adsorption capacities of N_0_PP, N_20_PP, N_40_PP, N_60_PP, and N_80_PP hydrogels for Cu^2+^ reached 258.38, 225.30, 261.60, 252.33, and 257.12 mg/g, respectively, while the adsorption percentages for MB within 5 h were 75.98%, 69.57%, 77.62%, 82.97%, and 88.18%. More importantly, the incorporation of nanoclay significantly enhanced the mechanical properties of the N_x_PP hydrogel adsorbents, increasing the compressive strength from 89.87 kPa (N_0_PP) to 560.49 kPa (N_80_PP). This excellent mechanical performance imparts reusability to the N_x_PP hydrogels, with adsorption capacity remaining high after five adsorption–desorption cycles. The adsorption kinetics followed a pseudo-second-order model, and the adsorption isotherms fit the Freundlich model. While this study focused on single-metal systems to establish baseline adsorption capacities, the influence of co-existing ions in multi-component wastewater remains an important consideration for real-world applications. Future work will systematically investigate competitive adsorption behavior, selectivity, and interference mechanisms in complex matrices containing multiple heavy metal ions, further advancing the practical utility of N_x_PP hydrogels. In conclusion, the N_x_PP hydrogel adsorbents demonstrate great potential for the removal of heavy metal ions and organic dyes from wastewater.

## 4. Materials and Methods

### 4.1. Experimental Reagents

γ-PGA (95%, molecular weight: ~1000 kDa) was purchased from Shanghai Yika Biotechnology Co., Ltd. (Shanghai, China). PEI (Mw ~70,000, 30% in water) was bought from Adamas Beta (Shanghai, China). Cu(NO_3_)_2_·3H_2_O (Analytical Reagent, AR), FeCl_3_·6H_2_O (AR), zinc chloride (AR), EDC (purity ≥ 98%) and NHS (purity ≥ 98%) were purchased from Shanghai Aladdin Biochemical Technology Co., Ltd. (Shanghai, China). MB (AR) was obtained from Shanghai Titan Technology Co., Ltd. (Shanghai, China). Laponite nanoclay was purchased from Zhejiang Institute of Geologic and Mineral Resources (Hangzhou, China).

### 4.2. Preparation of γ-PGA/PEI (P_x_P) and N_x_PP Hydrogels

To prepare P_x_P hydrogels (x represents the weight of γ-PGA, specifically: 0.1 g, 0.15 g, and 0.2 g), γ-PGA in quantities of 0.1 g, 0.15 g, and 0.2 g was dissolved in 1.6 mL of deionized water under magnetic stirring. Then, 0.2 mL of PEI was added to the above γ-PGA solution and stirred until homogenous. After that, 0.04 g of EDC/NHS was dissolved in 0.4 mL of deionized water and added to the above solution to initiate gelation, resulting in the formation of P_0.1_P, P_0.15_P, and P_0.2_P hydrogels. To prepare N_x_PP hydrogels, 0, 20, 40, 60, and 80 mg of nanoclay was dispersed in 1.6 mL of deionized water under magnetic stirring. Then, 0.2 g of γ-PGA powder was added to the nanoclay dispersion and stirred until homogeneous. Subsequently, 0.2 mL of PEI solution was added to the γ-PGA-nanoclay dispersion and stirred until fully mixed. Finally, 0.04 g of EDC/NHS was dissolved in 0.4 mL of deionized water and added to the solution to initiate the gelation, forming N_0_PP, N_20_PP, N_40_PP, N_60_PP, and N_80_PP hydrogels.

### 4.3. Characterizations

To observe the microstructure, the prepared hydrogels were frozen at −80 °C for 24 h and then freeze-dried to obtain lyophilized samples. These samples were examined using SEM (Zeiss Sigma 300, Carl Zeiss AG, Oberkochen, Germany). Elemental mapping of the hydrogel samples was performed using EDS coupled with the SEM. FTIR spectroscopy of hydrogel samples was studied using an infrared spectrometer (NICOLET-380, Thermo Fisher Scientific, Waltham, WA, USA). X-ray photoelectron spectroscopy (XPS, Thermo Kalpha, Thermo Fisher Scientific, Waltham, WA, USA) was used to determine the binding energies of elements.

### 4.4. Swelling Performance of N_x_PP Hydrogels

The swelling of the hydrogels was evaluated by monitoring the weight changes over time. Specifically, 0.03 g (W_0_) of freeze-dried P_0.1_P, P_0.15_P, and P_0.2_P hydrogels were placed into sealed vials containing 10 mL of deionized water. At predetermined time intervals (3, 5, 10, 20, and 30 min), the hydrogels were removed, and surface water was blotted with filter paper. The hydrogels were then weighed, and the weight at time, t, was recorded as W_t_. The swelling ratio of N_x_PP hydrogels was determined similarly, with 0.05 g (W_0_) of freeze-dried N_0_PP, N_20_PP, N_40_PP, N_60_PP, and N_80_PP hydrogels placed in sealed vials containing 10 mL of deionized water. After the predetermined time intervals (3, 5, 10, 20, and 30 min), the hydrogels were removed, blotted dry, and weighed (W_t_). All experiments were performed in triplicate. The water absorption (WA, g/g) was calculated using Equation (1), and the swelling kinetics curves were plotted.

WA (g/g) = W_t_ − W_0_/W_0_ (1)

### 4.5. Mechanical Study of N_x_PP Hydrogels

The mechanical properties of hydrogels were investigated using a universal testing machine (TS-2632, Hongpu Technology (Hong Kong, China) Co., Ltd.). Hydrogels were molded into cylindrical samples with a diameter of 8 mm and a height of 10 mm. The preset strain rate was 20 mm/min, with a strain range of 0–90%. The tests were conducted at 25 °C, and the hydrogels were compressed to 80% of their initial height.

### 4.6. Adsorption Kinetics of P_x_P Hydrogels

Cu(NO_3_)_2_ was dissolved in deionized water to prepare Cu^2+^-containing wastewater. To investigate the Cu^2+^ adsorption capacity of P_x_P hydrogels, 30 mg of freeze-dried hydrogel was placed into a centrifuge tube containing 30 mL of Cu^2+^-containing wastewater (300 mg/L). The tube was then shaken at 150 rpm at room temperature for 30 min. At predetermined time points, the centrifuge tubes were removed, and the hydrogels were discarded. All experiments were conducted in triplicate. The concentration of Cu^2+^ in the wastewater was measured using inductively coupled plasma mass spectrometry (ICP-MS, Avio 200, PerkinElmer, Hopkinton, MA, USA).

### 4.7. Adsorption Kinetics of N_x_PP Hydrogels

To study the adsorption kinetics of N_x_PP hydrogels, we examined the adsorption capacity over different time intervals. A 30 mg sample of freeze-dried N_x_PP hydrogels was placed into centrifuge tubes containing 30 mL of Cu^2+^-containing wastewater (300 mg/L). The tubes were shaken at 150 rpm at room temperature for various times (5, 10, 15, 20, 25, 30, 35, and 40 min). At each time point, the tubes were removed, and the hydrogels were discarded. All experiments were conducted in triplicate. The concentration of Cu^2+^ in the wastewater was measured using inductively coupled plasma mass spectrometry (ICP-MS, Avio 200, PerkinElmer, Massachusetts, USA).

### 4.8. Effect of pH on the Adsorption Capacity of N_x_PP Hydrogels

To investigate the effect of pH on the adsorption capacity of N_x_PP hydrogels, we adjusted the pH of the Cu^2+^-containing wastewater to 1.0, 3.0, and 6.0 using HCl. A 30 mg sample of freeze-dried hydrogels was added to centrifuge tubes containing 30 mL of Cu^2+^-containing wastewater (300 mg/L) at different pH levels. The tubes were shaken at 150 rpm at room temperature for 30 min. At the designated time points, the tubes were removed, and the hydrogels were discarded. The concentration of Cu^2+^ in the wastewater was measured using inductively coupled plasma mass spectrometry (ICP-MS, Avio 200, PerkinElmer, Massachusetts, USA). All experiments were performed in triplicate.

### 4.9. Effect of Temperature on the Adsorption Capacity of N_x_PP Hydrogels

The effect of temperature on the adsorption capacity of N_x_PP hydrogels was evaluated by placing 30 mg of freeze-dried N_x_PP hydrogels into centrifuge tubes containing 30 mL of Cu^2+^-containing wastewater (300 mg/L). The tubes were immediately transferred to shaking incubators set at different temperatures (293 K, 303 K, and 313 K) and shaken at 150 rpm for 30 min. After the predetermined time, the tubes were removed, and the hydrogels were discarded. The concentration of Cu^2+^ in the wastewater was measured using inductively coupled plasma mass spectrometry (ICP-MS, Avio 200, PerkinElmer, Massachusetts, USA). Each experiment was repeated in triplicate.

### 4.10. Adsorption Study of N_x_PP Hydrogels for Different Metal Ions

To assess the adsorption capacity of N_x_PP hydrogels for different metal ions, we prepared Fe^3+^ and Zn^2+^-containing wastewater (300 mg/L). Then, a 30 mg sample of freeze-dried N_x_PP hydrogels was placed into centrifuge tubes containing 30 mL of each metal ion-containing solution. The tubes were immediately transferred to a shaking incubator at room temperature and shaken for 30 min (150 rpm). After the designated time point, the tubes were removed, and the hydrogels were discarded. All experiments were conducted in triplicate. The concentration of Fe^3+^ and Zn^2+^ in the wastewater was measured using inductively coupled plasma mass spectrometry (ICP-MS, Avio 200, PerkinElmer, Massachusetts, USA).

### 4.11. Adsorption Capacity of N_x_PP Hydrogels for MB

We prepared an MB solution by dissolving 20 mg of MB in 1 L of deionized water to obtain a 20 mg/L solution. A 30 mg sample of freeze-dried N_x_PP hydrogels was placed into a centrifuge tube containing 30 mL of MB solution (20 mg/L). The tubes were shaken at 150 rpm at room temperature for 3 and 5 h. At the designated time points, the tubes were removed, and the solutions were filtered using a 0.45 µm filter. The adsorption capacity of the hydrogels for MB dye was determined by measuring the dye concentration in the prepared wastewater using a UV-visible spectrophotometer (N4/N4S INESA, Jinan Saichang Scientific Instrument Co., Ltd., Jinan, China). Kinetic adsorption curves were plotted, and each experiment was performed in triplicate.

### 4.12. Adsorption Mechanism Study of N_x_PP Hydrogels

To investigate the adsorption mechanism of N_x_PP hydrogels, we analyzed the chemical composition of the hydrogels after adsorption using XPS. A 30 mg sample of freeze-dried N_x_PP hydrogels was placed in centrifuge tubes containing 30 mL of wastewater solutions (300 mg/L of Cu^2+^, Fe^3+^, and Zn^2+^, and 20 mg/L of MB). The tubes were shaken at 150 rpm at room temperature for 30 min. After adsorption, the hydrogels were freeze-dried, ground into powder, and analyzed using XPS.

### 4.13. Recovery and Reusability Study of N_x_PP Hydrogels

N_x_PP hydrogels were placed in centrifuge tubes containing a Cu^2+^ solution of a specified concentration (mg/L). The centrifuge tubes were then transferred immediately to a shaking incubator and agitated at 150 rpm for 30 min at room temperature. At predetermined time points, the centrifuge tubes were removed from the shaking incubator, and the hydrogels were recovered. After each adsorption cycle, the hydrogels were placed in centrifuge tubes containing 5 mL of 1 mol/L HCl and subsequently agitated at 120 rpm for 2 h to facilitate the desorption. Following desorption, the hydrogels were removed and washed three times with deionized water, then freeze-dried for use in subsequent adsorption experiments. This process was repeated for a total of five cycles, with each experiment conducted in triplicate. The recovery and reusability were further compared as activity, which is defined as the adsorption capacity at a given cycle divided by the adsorption capacity in the first cycle.

### 4.14. Data Analysis

The adsorption amount (Q_e_) was calculated using the following equation (Equation (2)):

(2) $$Q_{e}=(C_{0}-C_{e})\times V/W$$

where Q_e_ (mg/g) represents the adsorption amount, C_0_ (mg/L) is the initial concentration of the solution, C_e_ (mg/L) is the concentration after adsorption, V (L) is the solution volume, and W (g) is the weight of the freeze-dried hydrogel.

The adsorption kinetics were described by pseudo-first-order (Equation (3)), pseudo-second-order (Equation (4)), and intraparticle diffusion (Equation (5)) models:

(3) $$\ln\left(Q_{e}-Q_{t}\right)=\ln Q_{e}-k_{1}t$$

(4) $$t/Q_{t}=t/Q_{e}+1/\left(k_{2}Q_{e}^{2}\right)$$

(5) $$Q_{t}=k_{i}\sqrt{t}+C$$

where Q_e_ (mg/g) is the adsorption amount at equilibrium, Q_t_ (mg/g) is the adsorption amount at time t (min), k_1_ (min^−1^) and k_2_ (g·mg^−1^·min^−1^) are the adsorption rate constants, k_i_ (mg·g^−1^·min^1/2^) is the intraparticle diffusion rate constant, and C is the intraparticle diffusion constant.

Adsorption thermodynamics were described by the Langmuir (Equation (6)), Freundlich (Equation (7)), and Dubinin–Radushkevich (Equation (8)) models:

(6) $$1/Q_{e}=1/Q_{\max}+\left(1/\left(K_{L}Q_{\max}\right)\right)\times 1/C_{e}$$

(7) $$\log Q_{e}=\log K_{F}+\left(1/n\right)\times \log C_{e}$$

(8) $$\ln Q_{e}=\ln Q_{\max}-{\beta R}^{2}T^{2}{\left[\ln\left(1+1/C_{e}\right)\right]}^{2}$$

where Q_e_ (mg·g^−1^) is the adsorption amount at equilibrium, Q_max_ (mg·g^−1^) is the maximum adsorption capacity, K_L_ (L·g^−1^) and K_F_ [(mg·g^−1^)/(mg·L^−1^)^n^] are constants for the Langmuir and Freundlich models, respectively, n is a constant representing adsorption intensity, C_e_ is the equilibrium concentration of metal ions, β (mol^2^·kJ^−2^) is a constant related to adsorption energy, R = 8.314 J·mol^−1^·K^−1^ is the gas constant, and T (K) is the temperature.

## Figures and Tables

**Table 1:** Kinetic parameters of pseudo-first-order and pseudo-second-order models for the adsorption of Cu^2+^ ions onto N_x_PP hydrogels.

Hydrogel	k_1_ (1/min)	Q_e_ (mg/g)	R^2^ (Pseudo-First-Order)	k_2_ (g/mg·min)	Q_e_ (mg/g)	R^2^ (Pseudo-Second-Order)
N_0_PP	0.12	65.4	0.970	0.0061	70.1	0.996
N_20_PP	0.14	70.3	0.975	0.0075	74.8	0.997
N_40_PP	0.16	75.6	0.981	0.0088	78.9	0.991
N_60_PP	0.18	78.7	0.984	0.0102	81.3	0.998
N_80_PP	0.19	79.8	0.980	0.0110	82.1	0.998

**Table 2:** Parameters of adsorption isotherm models for N_x_PP hydrogels.

Hydrogel	Q_max_ (mg/g)	K_L_ (L/mg)	R^2^ (Langmuir)	K_F_	n	R^2^ (Freundlich)	β	E (KJ/mol)	R^2^ (Temkin)
N_0_PP	95.4	0.015	0.991	8.4	2.3	0.999	0.00465	10.37	0.760
N_20_PP	102.6	0.017	0.991	9.2	2.5	0.996	0.00405	11.11	0.758
N_40_PP	110.2	0.020	0.992	10.6	2.7	0.991	0.00355	11.87	0.764
N_60_PP	115.8	0.022	0.989	11.8	2.9	0.997	0.00312	12.66	0.749
N_80_PP	118.7	0.025	0.969	12.4	3.0	0.989	0.00282	13.32	0.705

**Table 3:** Recovery and reusability activity during each of the five cycles for the N_x_PP hydrogels.

Cycle	N_0_PP (%)	N_20_PP (%)	N4_0_PP (%)	N_60_PP (%)	N_80_PP (%)
1	100.0 ± 1.3	100.0 ± 1.2	100.0 ± 1.1	100.0 ± 1.0	100.0 ± 0.9
2	97.3 ± 1.5	97.9 ± 1.4	98.1 ± 1.3	98.4 ± 1.1	98.6 ± 1.0
3	95.6 ± 1.6	96.4 ± 1.5	97.3 ± 1.3	97.7 ± 1.2	98.1 ± 1.0
4	93.8 ± 1.7	95.1 ± 1.6	96.2 ± 1.4	96.8 ± 1.2	97.5 ± 1.1
5	92.0 ± 1.8	94.2 ± 1.7	95.3 ± 1.5	96.1 ± 1.3	96.9 ± 1.2

## Data Availability

The data presented in this study are available on request.

## Supplementary Materials

The following supporting information can be downloaded at: https://www.mdpi.com/article/10.3390/gels11040287/s1, Figure S1: FTIR spectra of N_x_PP hydrogels.
