# Supplementary material for: Development of a Novel Nanoclay-Doped Hydrogel Adsorbent for Efficient Removal of Heavy Metal Ions and Organic Dyes from Wastewater
Source: Gels. 2025 Apr 14;11(4):287. doi: 10.3390/gels11040287 (PMC12026840; doi:10.3390/gels11040287)
Supplement: Supplementary file 1 [file gels-11-00287-s001.zip › gels-3577529-supplementary.pdf]

Supporting information of

Development of a Novel Nanoclay-Doped Hydrogel Adsorbent for Efficient Removal of Heavy Metal Ions and Organic Dyes from Wastewater

Hang Zhao [1,†], Mengmeng Xie [2,†], Siyu He [1], Saishi Lin [1], Shige Wang [2,*] and Xiuying Liu [1,3,*]

1 School of Chemistry and Chemical Engineering, Wuhan Textile University, Wuhan 430200, China
2 School of Materials and Chemistry, University of Shanghai for Science and Technology, No. 334 Jungong Road, Shanghai 200093, China
3 Key Laboratory of Textile Fiber and Products, Ministry of Education, Wuhan Textile University, Wuhan 430200, China
* Correspondence: sgwang@usst.edu.cn (S.W.); liuxiuying@wtu.edu.cn (X.L.)
† These authors contributed equally to this work.

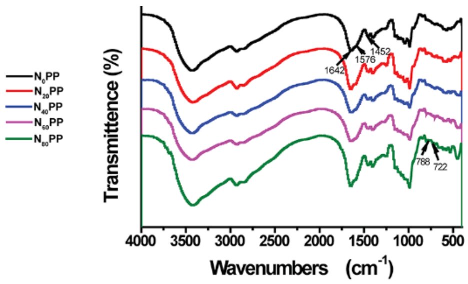

Figure S1. FTIR spectra of N_xPP hydrogels.
